# Targeting multiple health risk behaviours among vocational education students using electronic feedback and online and telephone support: protocol for a cluster randomised trial

**DOI:** 10.1186/s12889-015-1898-8

**Published:** 2015-06-13

**Authors:** Flora Tzelepis, Christine L Paul, John Wiggers, Kypros Kypri, Billie Bonevski, Patrick McElduff, Mary Ann Hill, Philip J Morgan, Marita Lynagh, Clare E Collins, Elizabeth Campbell, Ryan J Courtney, Kathy Chapman, Luke Wolfenden, Ashleigh Guillaumier, Andrew Searles

**Affiliations:** School of Medicine and Public Health, University of Newcastle, Callaghan, New South Wales, 2308 Australia; Hunter New England Population Health, Hunter New England Area Health District, New South Wales, Australia; Hunter Medical Research Institute, Newcastle, New South Wales, Australia; Hunter Institute of Technical and Further Education (TAFE), TAFE New South Wales, New South Wales, Australia; School of Education, University of Newcastle, Newcastle, New South Wales, Australia; School of Health Sciences, Faculty of Health and Medicine, University of Newcastle, New South Wales, Australia; National Drug and Alcohol Research Centre, University of New South Wales, New South Wales, Australia; Cancer Council New South Wales, Woolloomoolloo, New South Wales, Australia

**Keywords:** Cluster randomised trial, Multiple health risk behaviours, Smoking, Alcohol, Fruit, Vegetables, Physical activity, Vocational education

## Abstract

**Background:**

Technical and Further Education (TAFE) colleges are the primary provider of vocational education in Australia. Most TAFE students are young adults, a period when health risk behaviours become established. Furthermore, high rates of smoking, risky alcohol consumption, inadequate fruit and vegetable intake and insufficient physical activity have been reported in TAFE students. There have been no intervention studies targeting multiple health risk behaviours simultaneously in this population. The proposed trial will examine the effectiveness of providing TAFE students with electronic feedback regarding health risk behaviours and referral to a suite of existing online and telephone services addressing smoking, risky alcohol consumption, fruit and vegetable intake, and physical activity levels.

**Methods/Design:**

A two arm, parallel, cluster randomised trial will be conducted within TAFE campuses in New South Wales (NSW), Australia. TAFE classes will be randomly allocated to an intervention or control condition (50 classes per condition). To be eligible, students must be: enrolled in a course that runs for more than 6 months; aged 16 years or older; and not meet Australian health guideline recommendations for at least one of the following: smoking, alcohol consumption, fruit and/or vegetable intake, or physical activity. Students attending intervention classes, will undertake via a computer tablet a risk assessment for health risk behaviours, and for behaviours not meeting Australian guidelines be provided with electronic feedback about these behaviours and referral to evidence-based online programs and telephone services. Students in control classes will not receive any intervention. Primary outcome measures that will be assessed via online surveys at baseline and 6 months post-recruitment are: 1) daily tobacco smoking; 2) standard drinks of alcohol consumed per week; 3) serves of fruit consumed daily; 4) serves of vegetables consumed daily; and 5) metabolic equivalent minutes of physical activity per week.

**Discussion:**

Proactive enrolment to existing online and telephone services has the potential to address modifiable determinants of disease. This trial will be the first to examine a potentially scalable intervention targeting multiple health risk behaviours among students in the vocational training setting.

**Trial Registration:**

Australian New Zealand Clinical Trials Registry ACTRN12615000105549; Registered 5/2/15

## Background

Tobacco use, risky alcohol consumption, inadequate fruit and vegetable intake and insufficient physical activity are all modifiable risk factors of chronic diseases [1]. Young adulthood is a period when smoking becomes established [2], risky alcohol consumption increases [2], and fruit and vegetable intake [3] and physical activity declines [4]. Australian health guidelines recommend that adults: do not use tobacco [5]; consume no more than two standard alcoholic drinks per day (to reduce life-time disease risk) and no more than four standard alcoholic drinks on any one occasion (to reduce the risk of injury and acute problems) [6]; eat at least two serves of fruit and five serves of vegetables each day [7]; and do at least 150 min of moderate physical activity or at least 75 min of vigorous physical activity each week [8].

Technical and Further Education (TAFE) colleges are the primary national provider of vocational education and training in Australia and deliver nationally recognised training to students [9]. TAFE provides training for diverse career pathways including for example hairdressing, beauty therapy, fashion, automative, plumbing, electronics and commercial cookery [9]. There are approximately 1.8 million TAFE student enrolments per year nationally [10], and a high proportion of students are aged 15–34 years [9]. TAFE students have high rates of daily smoking (22 %), risky alcohol consumption (49 %), insufficient fruit (50 %) and vegetable (96 %) intake, inadequate physical activity (88 %) and almost all students (98 %) report two or more of these behaviours [11].

Systematic reviews have demonstrated that online programs and telephone services are each effective at reducing smoking rates [12, 13], and risky alcohol consumption [14, 15], and increasing healthy eating [16, 17], and physical activity [18, 19]. Online [20] and telephone interventions [21] are also effective at improving multiple health risk behaviours. A community randomised trial in the US with 3,391 adults found that online and telephone support significantly improved physical activity and also reduced the total number of health risk behaviours (included smoking, alcohol consumption, fruit and vegetables intakes) at 6-months follow-up [22]. In another trial with 3,344 US adults online intervention was found to significantly increase daily fruit and vegetable consumption and weekly physical activity, 6-months after the intervention, however there was no effect on smoking cessation [23].

Existing online and telephone services that are widely available to the community typically involve recruitment through mass media relying on individuals to initiate contact, an approach that limits their reach [24]. Proactive recruitment methods that involve the recruiter initiating contact with the individual have been shown to increase the use of telephone services [25]. For example, a population-based randomised controlled trial found that 52 % of smokers who were proactively offered quitline telephone callbacks accepted [26, 27] which is substantially higher than the 3.6 % of Australian smokers who initiate contact with the quitline on their own each year [24]. Further, 23 % of individuals proactively offered calls from the Get Healthy Information and Coaching telephone service, accepted the offer of assistance to improve their nutrition and physical activity levels [28], compared with the <1 % of individuals who inititate contact with this service each year [29]. Proactive enrolment has significant potential for improving the reach of existing services, and hence contributing to population level reductions in the prevalence of modifiable health risk behaviours.

Despite TAFE students having high rates of smoking, risky alcohol consumption, insufficient fruit and vegetable intake, and inadequate physical activity [11], no prior studies have assessed the effectiveness of either telephone-based or online programs with this population. Furthermore, no study has proactively and simultaneously offered both online and telephone support for all the health risk behaviours. The aim of this cluster randomised trial is to examine the effectiveness of providing TAFE students with electronic feedback and referral to a suite of online and telephone services to address their smoking, risky alcohol consumption, fruit and vegetable intake, and physical activity levels.

## Methods/Design

### Design

**Fig. 1:**
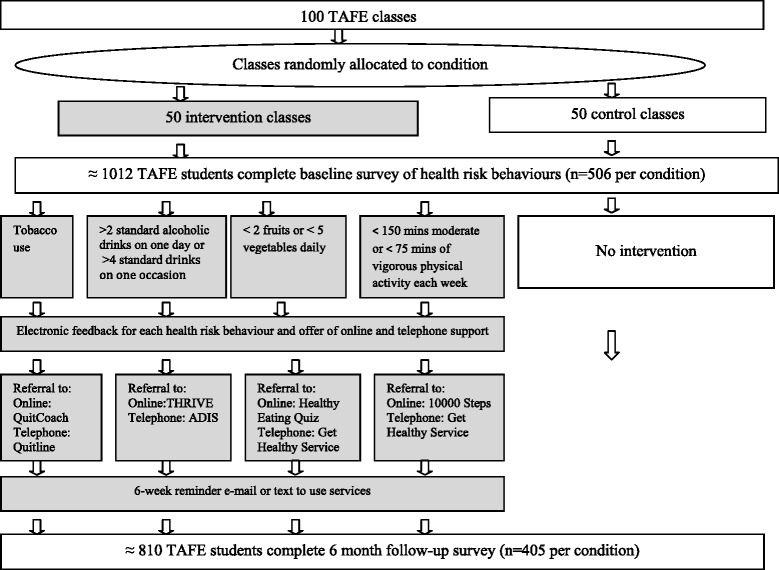
Flowchart of recruitment, intervention and follow-up

A two arm, parallel, cluster randomised trial will be conducted with 100 TAFE classes (50 per condition) at TAFE campuses in the Hunter Region of New South Wales Australia (Fig. 1). The CONSORT statement will be followed [30]. TAFE classes will be randomly allocated to either the intervention or control condition. In intervention classes, students will receive electronic feedback about their health risk behaviours and will be proactively referred to evidence-based online and telephone services for behaviours that do not meet Australian health guidelines. Students in control classes will not receive an intervention. Online surveys will occur at baseline and 6 months post-recruitment and will be administered in classrooms using a computer tablet.

### Setting and participants

TAFE campuses located in the Hunter Region (population 620,530) [31] will participate and TAFE records will be used to identify classes that run for >6 months. Eligible students will: be currently enrolled in a course that runs for >6 months; aged 16 years or older; and not meet Australian health guidelines for at least one of the risk behaviours as assessed by the baseline survey: smoking, risky alcohol consumption, insufficient fruit intake, inadequate vegetable intake, or insufficient physical activity.

### Randomisation

The randomisation sequence will be generated by an independent statistician using a random number function in Microsoft Excel. An equal number of classes will be randomised to each condition (1:1) using a randomised block design, with a block size of six, to ensure the conditions are balanced. Participant blinding will not be possible because they will be aware of the trial’s conditions and the differences will be apparent.

### Recruitment and baseline survey

An information letter describing the trial and intervention and control conditions will be distributed to students. A week later, students will be provided with a verbal explanation of the study in class and invited to complete the baseline survey using a computer tablet. Informed consent will be recorded via the first page of the online baseline survey which will explain that if the student would like to take part in the trial they should choose ‘Next’ and if they do so they agree to participate in the trial. Across the 100 classes, approximately 1,100 eligible TAFE students will be approached, and assuming the 92 % response fraction achieved in pilot work, we expect 1,012 students to be recruited into the trial and complete the baseline survey. The baseline survey will include standard and validated questions to examine the student’s current smoking, alcohol consumption, daily fruit and vegetable intake, and physical activity. Students’ knowledge of the Australian guidelines for each risk behaviour and intentions to change behaviours that do not meet Australian guidelines will be assessed. Socio-demographic characteristics, self-reported height, self-reported weight, and type of TAFE course students are studying will also be collected at baseline.

## Intervention condition

### Electronic feedback, online and telephone support

Online support and telephone support strategies were chosen based on the APEASE criteria for selecting interventions: Affordability, Practicability, Effectiveness and cost-effectiveness, Acceptability, Safety and Equity [32]. The online and telephone services: are available at no cost to participants; have potential for broad reach; and are supported by systematic reviews showing that online and telephone services using evidence-based behaviour change techniques such as motivational interviewing, monitoring and tailored feedback are effective across a range of behaviours and population groups [12-19].

The intervention will involve the following components (see Fig. 1).

#### Electronic health risk feedback

Based on their responses to the baseline survey, students in the intervention classes will receive immediate electronic feedback via the computer tablet relating to the reported behaviours that do not meet Australian guidelines. The feedback will include information about the relevant evidence-based guideline and advice to assist behaviour change. For instance, students who consume <2 serves of fruits or <5 serves of vegetables each day will receive immediate feedback that they are not meeting the Australian Guide to Healthy Eating nutrition recommendations, and that healthy eating can improve their overall health. They will then be informed via the computer tablet that online and telephone services and advice from a dietitian or doctor can help increase their fruit and vegetable intake.

#### Referral to online and telephone services

Following the electronic feedback, the software program will offer referral to existing online and telephone services for each behaviour where the participant does not meet the Australian guidelines. These services will be provided at no cost to participants. Students who agree to use online programs will be asked to provide their e-mail or mobile phone details and the hyperlink for the relevant program(s) will be sent to them. Students who agree to use telephone services will be asked to provide their contact details including a home and/or mobile phone number. Electronic referral will be sent to the NSW Quitline and the Get Healthy Information and Coaching Service and within two days of referral these telephone services will contact the student directly to provide advice and support. Given the Alcohol Drug Information Service (ADIS) is an anonymous service, participants will be sent the ADIS telephone number via e-mail or text so they can contact the service directly. Students will be able to accept either or both the online and telephone services for any behaviour that does not meet recommended Australian guidelines.

### Online programs

#### Smoking – QuitCoach program

The QuitCoach (http://www.quitcoach.org.au) provides individually tailored advice and support using principles of cognitive behavioural therapy to help smokers quit [33]. The QuitCoach creates a two to four page personalised quit plan by asking participants a series of questions about their current smoking, plans to quit, motivation, confidence and past quit attempts. Intervention elements include: strategies to help resist urges to smoke, deal with nicotine withdrawal, and situations where the individual used to smoke. The program permits unlimited access and provides evidence-based advice and information sheets and sends reminder emails to the participant to return to the QuitCoach program to review their current situation. For instance, if the participant has quit smoking, QuitCoach will make adjustments to their personalised plan to provide relapse prevention strategy advice that reflects the progress made.

#### Alcohol – THRIVE

Tertiary Health Research Intervention Via E-mail (THRIVE) is an online alcohol screening and brief intervention program (https://thrivehealth.org.au/curtin/survey.php) developed to reduce risky drinking [34]. A randomised trial involving 2,435 Australian university students who did not seek help themselves showed that 6 months after intervention, participants in the THRIVE condition drank 9 % less often, 7 % less alcohol per occasion and 11 % less alcohol overall compared to controls [34]. The program involves a 5–10 min session of assessment and personalised feedback. The program examines the participant’s drinking patterns, dependence symptoms, and alcohol-related problems. Based on participant responses THRIVE provides feedback about: 1) classification of the participant’s drinking as moderate, hazardous, harmful or possibly alcohol dependent; 2) associated health risk and strategies for reducing alcohol consumption; 3) the participant’s drinking compared to other people of the same age and gender; 4) an estimated blood alcohol concentration for their heaviest drinking occasion in the past four weeks; and 5) their estimated expenditure on alcohol in the last year.

#### Fruit and vegetable intake – The Healthy Eating Quiz

The Healthy Eating Quiz is an online program (http://healthyeatingquiz.com.au) developed to identify alignment of current eating habits with the Australian Dietary Guidelines. It provides automated real-time feedback on components where improvements are needed. The Healthy Eating Quiz takes 5 min to complete online and examines the frequency and variety of nutrient-dense core foods including fruit and vegetables. At the end of the quiz the participant receives an overall Australian Recommended Food Score as an indicator of overall diet quality. Suggestions for improving diet quality are also provided based on responses to the quiz. The Australian Recommended Food Score has been validated in children, adolescents and adults with higher scores associated with more optimal nutrient intake profiles [35, 36].

#### Physical Activity – 10,000 Steps program

The 10,000 Steps online program (http://www.10000steps.org.au) encourages participants to use a pedometer to record their daily step counts on a personal step log, indicate their goal steps and monitor progress toward increasing physical activity [37]. Participants may also: participate in individual walking challenges; add virtual walking buddies, allowing them to share information about their daily step counts; share information about their daily step counts; and access and contribute to online discussions [37]. An online library is also embedded in the program that includes information about physical activity and healthy lifestyles, and the national physical activity guidelines.

### Telephone services

#### Smoking – NSW Quitline

The NSW Quitline is a population-wide service offering multiple quitline-initiated telephone counselling calls to smokers who self refer or are referred to the Quitline by healthcare professionals [25]. The NSW Quitline uses cognitive behaviour therapy and motivational interviewing techniques to support smokers to quit. The NSW Quitline offers six relapse-responsive proactive telephone counselling calls to smokers on the initial call date; on the quit date; and at 3, 7, 14, and 30 days after the quit date. The NSW Quitline counselling calls include: identifying and coping with smoking triggers; information on effective quitting aids; advice on setting tasks to assist with quitting; and promotion of self-efficacy and relapse prevention strategies.

#### Alcohol - Alcohol Drug Information Service (ADIS)

The NSW ADIS is an anonymous telephone service providing callers with: information about their alcohol consumption and associated risks; advice about effective interventions for reducing or stopping alcohol use; counselling tailored to the participant’s needs; and, if required, a referral for treatment. The ADIS counsellors provide personalised advice about the short-term and long-term effects of alcohol use, signs of intoxication and alcohol dependence, effective interventions for reducing alcohol intake and strategies for dealing with situations that trigger risky drinking.

#### Fruit and vegetable intake and physical activity – Get Healthy Information and Coaching Service

The Get Healthy Information and Coaching Service provides telephone advice regarding fruit and vegetable intake and physical activity [38]. The service offers 10 coaching telephone calls with most calls occurring within 12 weeks. During the initial call, the health coach will spend up to 20 min talking to the participant about the changes they wish to make to become healthier, their current nutrition and physical activity levels, and will ask the participant how much they weigh and their waist measurement. The subsequent coaching calls last 10–15 min and include: setting nutrition and/or physical activity goals; developing strategies for eating healthily; advice about how to incorporate sufficient fruit, vegetables and physical activity into daily life; and enhancing motivation [39]. Strategies for overcoming difficulties in achieving the participant’s nutrition and/or physical activity goals are also discussed.

### Reminder e-mails or texts

Six weeks after the baseline survey, personalised e-mails or texts will be sent to remind intervention group participants about the online and/or telephone services they agreed to use in the baseline survey. The messages will encourage students to use the online program(s) and will include the relevant hyperlinks to the program(s). Students who were referred to a telephone service will be asked if they received calls from the relevant service(s) and if calls have been completed if they would like to be re-referred to the service for additional support.

### Control condition

Students in the control classes will be asked to complete the baseline and follow-up surveys but will not receive an intervention.

### Follow-up survey

The follow-up survey will be administered via computer tablet to the same students while in class, 6 months after recruitment. Based on TAFE records of student retention in courses, it is expected that the trial’s retention rate will be about 80 %, which will result in approximately 810 students (405 per condition) completing the 6-month assessment. The students’ sex, year of birth, TAFE class, and first and last letters of mother’s first name will be used to link baseline and follow-up survey data. The probability of a mismatch using this procedure has been calculated to be <1 in 148 000 [40]. The 6-month assessment will examine TAFE students’ current smoking, alcohol consumption, fruit and vegetable intakes and physical activity, knowledge of Australian health guidelines and intentions regarding changing health risk behaviours.

### Primary outcome measures

#### Smoking

Two items will measure students’ daily smoking. TAFE students will be asked *“Do you currently smoke any tobacco products?”* and will select from the response options *daily, at least once a week, less often than once a week,* and *not at all.* Students will also be asked *“Would you have smoked at least 100 cigarettes or the equivalent amount of tobacco in your life?”* [41]. These are the standard items used to measure the prevalence of smoking in the Australian population [41].

#### Fruit and vegetable intake

The National Health Survey items will be used to determine usual daily fruit and vegetable intake: *1) How many serves of fruit do you usually eat each day?* One serve of fruit is one medium sized piece of fruit (e.g., apple), two small pieces (e.g., apricots), one cup chopped or canned fruit*;* and *2) How many serves of vegetables do you usually eat each day?* One serve of vegetables is half a cup of cooked vegetables, one medium potato or one cup salad vegetables [42].

#### Alcohol

Weekly alcohol consumption will be assessed by asking how many standard drinks participants consumed on each of the previous seven days beginning with yesterday. A picture showing a standard drinks chart, plus the number of standard drinks in some bulk alcohol containers will accompany each question. This measure improves accuracy by providing a recall prompt and estimating actual rather than usual alcohol consumption [43].

#### Physical Activity

Physical activity will be measured using the modified version [44] of the Godin Leisure Time Exercise Questionnaire (GLTEQ) [45] which measures in the past month the average number of times per week a participant engages in mild, moderate or vigorous physical activity for 10 min or longer. As per the scoring rules for the GLTEQ, weights of 9, 5 and 3 will be applied for each of the vigorous, moderate and mild responses respectively [45]. Participant responses for each category will be converted into metabolic equivalent scores (i.e., MET-minutes/week) by multiplying the weekly minutes of: mild activity by 2.5 METs; moderate activity by 4 METs; and vigorous activity by 7.5 METs [46].

### Secondary outcomes

#### Knowledge of recommended Australian guidelines

Participants’ knowledge of the recommended Australian guidelines for each risk behaviour will be explored. For smoking, alcohol consumption, fruit and vegetable intake, and physical activity, participants will be asked to select from a list of categories the option that reflects the relevant current Australian guideline.

#### Intentions to change health risk behaviours

Participants who do not meet the recommended guidelines for each risk behaviour will be asked whether they intend to improve this behaviour in the future. The timeframes for improving the behaviour that participants will be able to choose from will be *in the next 30 days, in the next 6 months, may in the future but not in the next 6 months* and *never*.

#### Body Mass Index (BMI)

To calculate BMI, participants will be asked to report their height in centimetres and their weight in kilograms. Conversion tables will accompany these questions so that participants can convert their height from feet and inches into centimetres and their weight from stone into kilograms if needed.

#### Sociodemographic characteristics

Sociodemographic characteristics such as age, sex, country of birth, Aboriginal or Torres Strait Islander origin, marital status, employment status and highest level of education will be recorded at baseline.

### Process measures

During the baseline survey, the computer software will record the number of intervention group participants who elect to be sent the hyperlink for each online program and referred to each telephone service. As part of the 6-month follow-up survey the number of intervention group participants who report using each online program and/or telephone service in the past 6 months and exposure to these interventions will be determined.

### Statistical analysis

Descriptive statistics will be used to present baseline characteristics for the intervention and control groups. Logistic regression will be used to test if there is a statistically significant difference in the change in prevalence of daily smoking between the two treatment groups. The outcome in the model will be smoking status and the predictors will include time, group and an interaction term between time and group. The *p*-value associated with the interaction term will be used to determine if there was a statistically significant difference in change between the groups. Linear regression will be used to test if there was a statistically significant different change in the mean level of the other outcomes between the groups. The outcome in each model will be the participant’s value of the outcome measure post treatment and the predictors will include their baseline value and treatment group (i.e., Analysis of Covariance). The p-value associated with treatment group will be used to determine if there was a statistically significant difference in the change from baseline to follow-up between the groups. Both the logistic and linear regression models will also be fit within a Generalised Estimating Equation framework to adjust for the correlation of responses within classes. All analyses will be conducted according to the Intention To Treat principle and the approach suggested by White and colleagues will be used to handle missing data [47]. The initial analysis will include all available data and will be valid under the assumption that the missing data are missing completely at random. Sensitivity analyses will be conducted under different assumptions about the missing data mechanism.

### Sample size

As there are five primary endpoints (smoking, alcohol consumption, fruit intake, vegetable intake and physical activity) an alpha level of 0.01 will be used to adjust for the multiple comparisons. The prevalence of daily smoking in the control group at follow-up is expected to be 22 % [11]. We aim to recruit 1012 TAFE students at baseline (i.e., 506 per condition). Allowing for 20 % loss to follow-up at 6 months, data will be available for 405 participants per condition. With an expected intra-cluster correlation coefficient (ICC) of 0.005 this will give the study a design effect of approximately 1.04 and an effective sample size of 389 per group, which will provide 80 % power to find an absolute difference of 9.5 % (in daily smoking rates) between the groups at the follow-up visit. Allowing for a higher ICC for the continuous outcomes (0.01), the study will have 80 % power to detect an absolute difference of 0.25 SD in these measures at the 1 % significance level, which equates to a difference of approximately 1.25 standard drinks of alcohol, 0.25 pieces of fruit, 0.25 vegetables and 50 MET minutes of physical activity per week.

### Ethics approval & trial registration

The University of Newcastle Human Research Ethics Committee has granted ethical approval for this cluster randomised trial (H-2014-0012). The trial is registered with the Australian New Zealand Clinical Trials Registry (ACTRN12615000105549).

## Discussion

This trial will be the first to target smoking, alcohol consumption, fruit and vegetable intake and physical activity in vocational education students using electronic feedback, online and telephone support. This trial will provide TAFE students with immediate electronic feedback and proactive referral to online and telephone services for each health behaviour that does not meet Australian health guidelines. Research has demonstrated that proactive enrolment into the NSW Quitline [26, 27] and the Get Healthy telephone service substantially increased service use [28]. This trial will extend this prior research by proactively and simultaneously offering online and telephone support for multiple health risk behaviours, an intervention with the potential to increase the population impact of these existing evidence-based services.

## Abbreviations

TAFE: Technical and Further Education
NSW: New South Wales
APEASE: Affordability, Practicability, Effectiveness and cost-effectiveness, Acceptability, Safety and Equity
ADIS: Alcohol Drug Information Service
THRIVE: Tertiary Health Research Intervention Via E-mail
GLTEQ: Godin Leisure Time Exercise Questionnaire
MET: Metabolic equivalent
BMI: Body Mass Index
ICC: Intra-cluster correlation coefficient
